# Spatio-Temporal Expression Pattern of CAKUT Candidate Genes *DLG1* and *KIF12* during Human Kidney Development

**DOI:** 10.3390/biom13020340

**Published:** 2023-02-09

**Authors:** Daniela Veljačić Visković, Mirela Lozić, Martina Vukoja, Violeta Šoljić, Katarina Vukojević, Merica Glavina Durdov, Natalija Filipović, Bernarda Lozić

**Affiliations:** 1 Paediatric Diseases Department, University Hospital of Split, Spinčićeva 1, 21 000 Split, Croatia; 2 Department of Anatomy, Histology and Embryology, University of Split School of Medicine, Šoltanska 2, 21 000 Split, Croatia; 3 Laboratory of Morphology, Department of Histology and Embryology, School of Medicine, University of Mostar, 88 000 Mostar, Bosnia and Herzegovina; 4 Faculty of Health Studies, University of Mostar, 88 000 Mostar, Bosnia and Herzegovina; 5 Department of Anatomy, University of Mostar, 88 000 Mostar, Bosnia and Herzegovina; 6 Center for Translational Research in Biomedicine, University of Split School of Medicine, 21 000 Split, Croatia; 7 Department of Pathology, University Hospital Split, 21 000 Split, Croatia; 8 School of Medicine, University of Split, Šoltanska 2, 21 000 Split, Croatia

**Keywords:** *DLG1*, *KIF12*, *α-tubulin*, *Ki-67*, kidney development

## Abstract

We aimed to investigate expression of the novel susceptibility genes for CAKUT, *DLG1* and *KIF12*, proposed by a systematic in silico approach, in developing and postnatal healthy human kidneys to provide information about their spatiotemporal expression pattern. We analyzed expression of their protein products by immunohistochemistry and immunofluorescence and quantified relative mRNA levels by RT-qPCR. Statistically significant differences in expression patterns were observed between certain developmental stages. Strong expression of DLG1 was observed in the developing kidney, with a gradual decrease from the first phase of kidney development (Ph1) until the third phase (Ph3), when most nephrons are formed; at later stages, the highest expression was observed in the tubules. KIF12 was highly expressed in the developing structures, especially in Ph1, with a gradual decrease until the postnatal phase, which would indicate a significant role in nephrogenesis. Co-localization of DLG1 and KIF12 was pronounced in Ph1, especially on the apical side of the tubular epithelial cells. Thereafter, their expression gradually became weaker and was only visible as punctate staining in Ph4. The direct association of *DLG1* with *KIF12* as control genes of normal kidney development may reveal their new functional aspect in renal tubular epithelial cells.

## 1. Introduction

The metanephros appears at about the fifth developmental week, and upon completion of its differentiation, forms the permanent kidney [1,2]. It develops through complex reciprocally inductive interactions between the metanephric mesenchyme (MM) and the ureteric bud (UB), which later gives rise to the collecting tubules and the basic renal architecture [3,4].

The tip of each collecting tubule induces the mesenchymal caps to form renal vesicles, which develop into nephric tubules consisting of Bowman’s capsules, proximal and distal tubules, and loops of Henle [5,6]. This leads to the development of nephrons with filtering glomeruli by 10th week of gestation, when urine begins to form in the fetus [7]. The formation of new nephrons—nephrogenesis—is completed by the 34th week of gestation, but the kidneys continue to differentiate and grow during all the fetal period [5,8]. The described nephrogenesis is divided into 4 developmental stages: the first phase (Ph1) takes place from the 5th to about the 14th week of development, the second phase (Ph2) begins in the 15th and ends in about the 20th to 22nd week of development, after which the kidney enters the third phase (Ph3) and finally, at around the 32nd to 36th week of development, phase four (Ph4) is set which continues well into adult life [3].

The complexity of the developmental process affects the occurrence of anomalies, and the renal and urinary tract are the most common form of malformations at birth [9] Congenital anomalies of the kidney and urinary tract (CAKUT) are embryonic disorders that occur during nephrogenesis and include a wide range of structural malformations in the kidney and outflow tract. Over 50 genes are identified as disease-causing for CAKUT [10]. Our knowledge is mostly derived from mouse models and human syndromes, but the majority of potential genetic causes remain yet to be identified [10] Understanding the underlying genetic basis of CAKUT is fundamental for the development of accurate genetic testing strategies, which can guide clinical decision-making and lead to better treatment and outcome [11,12].

The carriers of pathogenic copy number variants (CNVs) present the best cohort for identifying the most likely CAKUT phenotype triggers. In our previous work, a systematic in silico approach using bioinformatics resources and expression profiling in the developing human and mouse kidney was performed. Using this approach, we identified five high-level genetic drivers (*DLG1, EDA2R, KIF12, PCDH9, TRAF7*) and proposed *DLG1* and *KIF12* as novel susceptibility genes for human kidney and urinary tract malformations [13].

The polarity protein discs large 1 (DLG1) is a multidomain scaffolding protein, a member of the family of molecular scaffolding proteins named membrane-associated guanylate kinase (MAGUK). These proteins are localized at the membrane–cytoskeleton interface and are known for their modular organization. They are involved in structural roles and various cellular processes, like the regulation of signaling pathways in polarized epithelial cells. The gene that encodes this protein is located at chromosome band 3q29 [14]. During embryogenesis in mice, *DLG1* is expressed in epithelial, mesenchymal, neuronal, endothelial, and hematopoietic cells [15]. The disruption of the murine *Dlg1* gene results in craniofacial dysmorphogenesis [15], perturbed ureteric branching, and disrupted nephrogenesis in mice [16]. Hypoplasia of the kidney and ureter, megaureter, duplicated ureter, hydronephrosis, malposition of the gonads, and vaginal aplasia were found in *Dlg1* knock-out mice at various incidences [17]. The absence of the *Dlg1* gene results in the persistence of the common nephric duct and less apoptosis. It leads to a dysfunctional vesicoureteral junction and vesical–ureteral reflux, with consequent megaureter or hydronephrosis [18]. In humans, *DLG1* is expressed ubiquitously. Six patients with a 3q29 microdeletion encompassing 22 genes, including *DLG1,* had various mental and physical impairments, and one of them had a horseshoe kidney and hypospadia [19].

*KIF12* encodes the kinesin-related protein KIF12, which belongs to the kinesin superfamily proteins (KIFs). KIFs are involved in the transport of organelles, protein complexes, and mRNAs, but also in chromosome movements [20]. These functions include axonal transport, microtubule sliding during nuclear fusion or division, and chromosome segregation during meiosis and early mitosis [21,22]. Identification and classification of KIF proteins in the mouse revealed that Kif12 is dominantly expressed in the kidney [23]. The first report of the comprehensive characterization of *KIF12* in humans showed that the gene is located on chromosome 9q32 [24]. Mrug et al. showed the expression pattern of *Kif12* in mice in various nephron structures and developmental time points. *Kif12* encodes a primary cilia-associated protein. In mice, it has been identified as a candidate genetic modifier for severity of kidney disease in autosomal recessive polycystic kidney disease [25].

The understanding of the *DLG1* and *KIF12* expression patterns in the fetal and postnatal human kidney remains limited. In our study, we focus on *DLG1* and *KIF12* as CAKUT candidate genes in order to characterize their normal expression pattern during kidney development. Our study aimed to investigate the expression and localization of these proteins in developing and postnatal healthy human kidneys from the 13th developmental week to postnatal age, to determine the most critical developmental period for the occurrence of CAKUT. 

## 2. Materials and Methods

### 2.1. Tissue Procurement and Processing

Human fetal kidney specimens were obtained after spontaneous pregnancy loss at the 13th, 15th, 16th, 21st, 22nd, 24th, 28th, 29th, 35th, 37th, and 38th gestational weeks (13 samples total), and four postnatal kidneys (1 month, 1.5 years, 17 years, and 71-year-old patients, consecutively) after accidental deaths. Kidney tissue embedded in paraffin was collected from the Department of Pathology, University Hospital in Split. Fetal maturity was determined from medical records and external measurements (crown–rump and femur length, head, and abdominal circumference, O’Rahilly 1972). Two fetal kidneys belonged to Ph1 (13th week), six to Ph2 (15th to 24th week), three to Ph3 (28th to 35th week), and two to Ph4 (37th and 38th week). Postnatal kidneys were defined as phase five (Ph5), one of which was neonatal (1 month), two pediatric (1.5 and 17 years), and one adult (71 years).

Tissue samples were fixed with 4% neutral paraformaldehyde, dehydrated in graded ethanol dilutions, embedded in paraffin blocks, serially cut into 4 µm thick slides and mounted on glass slides. Proper tissue preservation was confirmed by standard hematoxylin and eosin staining of every tenth section of each tissue block. After examination, only well-preserved tissue was used in further experiments and any macerated or poorly maintained samples were discarded. 

### 2.2. Immunohistochemistry with Chromogenic Detection

Immunohistochemistry (IHC) was performed on Ultra Benchmark (Ventana Medical Systems, Inc. Oro Valley, AZ, USA) according to the manufacturer’s instructions. Primary antibodies anti-DLG1, anti-KIF12, anti-Ki-67, and anti- α-tubulin were used (Table 1). 

Single IHC was performed with anti-DLG1 and anti-KIF12, consecutively. Hematoxylin counterstain was not included, due to easier signal quantification later on. In double IHC, secondary antibodies were conjugated with enzymes which react with the chromogens 3,3’-diaminobenzidine (DAB) and naphthol phosphate-diazonium salt (Fast Red), respectively. The enzyme horseradish peroxidase (HRP) converted the substrate DAB into a brown precipitate, and the enzyme alkaline phosphatase (AP) converted Fast Red into red precipitate. Three representative kidney samples were double stained using 3 different combinations. Anti-DLG1 (chromogen Fast Red) was stained with anti-Ki-67 (chromogen DAB), anti-KIF12 (chromogen Fast Red) with anti-αtubulin (chromogen DAB), and anti-DLG1 (chromogen Fast Red) with anti-KIF12 (chromogen DAB) (Table 1). No staining was observed when primary antibodies were omitted.

### 2.3. Immunofluorescence

Representative tissue samples were used for immunofluorescence (IF), to better visualize subcellular localization of the positive signal, as we described previously [26]. Briefly, the tissue was deparaffinized in xylol and rehydrated in graded ethanol dilutions and distilled water. Antigen retrieval was done in citrate buffer for 20 min at 95 °C in a water steamer and gradually cooled to room temperature. Afterwards, protein blocking buffer (ab64226, Abcam, Cambridge, UK) was applied for 20 min to prevent non-specific staining. The slides were then incubated with anti-DLG1 and anti-KIF12 primary antibodies (Table 1) overnight in a humidity chamber. Next day, corresponding fluorescent-dye-labeled secondary antibodies were applied (Donkey Alexa Fluor 488 Anti-Mouse, 1:400; 711-545-152; Jackson ImmunoResearch, Newmarket, UK) for 2 h. Finally, the nuclei were stained using 4′,6-diamidino-2-phenylindole (DAPI) and then cover-slipped (Immuno-Mount, Thermo Shandon, Pittsburgh, PA, USA). No immunoreactivity was observed when primary antibodies were omitted from the protocol. Sections were examined by fluorescence microscope (Olympus BX61, Tokyo, Japan) equipped with digital camera (DP71) and images were consecutively captured at 40×.

### 2.4. Data Acquisition, Quantification, and Statistical Analysis of Area Percentages 

Microscopic analysis was performed on the VENTANA DP 200 high-resolution six-slide high-speed brightfield scanner (F. Hoffmann-La Roche, Basel, Switzerland). Images were taken at ×20 magnification and processed using Adobe Photoshop and ImageJ. Moderate to strong levels of cDAB/IHC were regarded as positive, while a lack of staining was regarded as negative. These levels corresponded with the pattern of staining observed in the IF method, which confirmed specificity. Due to higher sensitivity and stability of the signal, chromogenic images were used for quantitative evaluation.

Quantitative analyses of IHC slides were performed as previously described [27]. To quantify DLG1 and KIF12 protein product expression, 20 non-overlapping representative visual fields were captured. Brown cytoplasm staining marked DLG1 or KIF12, consecutively. In double stained slides, red and brown staining assigned the expression KIF12 and DLG1, consecutively. For cell quantitative evaluation of DLG1 and KIF12 in single IHC slides were used ImageJ software (National Institutes of Health, Bethesda, MD, USA) and Adobe Photoshop CS6 (Adobe Systems, San Jose, CA, USA). Figures were prepared for analysis using subtraction of the median filter and thresholding to measure the section percentage area covered by the positive signal.

IBM SPSS statistics (IBM SPSS statistics software, New York, NY, USA) was utilized for statistical analyses with the probability level of *p* < 0.05 being regarded as statistically significant. A one-way ANOVA test followed by Tukey’s post-hoc test was used to compare the immunoexpression in different phases of development.

### 2.5. RNA Isolation and RT-qPCR

RNA was isolated from 15 human kidney samples fixed in paraffin. Two samples from Ph5 were omitted in qPCR analysis due to a lack of material. Multiple 5µm thick tissue samples were placed in a tube, deparaffinized with xylene, and centrifuged at 14,000 rpm for 2 min. The obtained precipitate was further treated according to the attached manufacturer’s instructions GenElute™ FFPE RNA Purification Kit (Sigma-Aldrich, Taufkirchen, Germany). The protocol started with deparaffinization of the tissue archived in paraffin through a series of washes with xylene and ethanol, followed by digestion with the included proteinase K and the appropriate buffer A. Buffer RL and ethanol were added to the lysate, and the solution applied to a spin column. The bound RNA was washed from the columns with wash solution A. Buffer RL and ethanol were then added to the lysate, and the solution was applied to the spin column. The bound RNA was washed off the columns with wash solution A. We used the Qubit™ 4 Fluorometer (Thermo Fisher Scientific Inc., Waltham, MA, USA) for the quantitation of total RNA in each sample. The samples were diluted to match the lowest measured concentration (14.3 ng/µL).

After RNA isolation, the one-step SYBR^®^ Green RT-qPCR with MMLV & hot-start Taq DNA Polymerase kit (Sigma-Aldrich, Germany) designed for measurement of gene expression was used. The master mix containing RNA, selected forward and reverse primers (Table 2), SYBR^®^ Green Taq ReadyMix for Quantitative RT-PCR, MgCl2, Moloney Murine Leukemia Virus Reverse Transcriptase (M-MLV RT), and nuclease-free water was mixed in a 96-well plate. Primers used in the RT-qPCR method (Table 2.) were designed using Primer-BLAST software (NCBI, Bethesda, MD, USA), which uses Primer3 to design PCR primers and then BLAST to screen primers against a specific database to avoid primer pairs that might cause nonspecific amplifications. All samples were prepared in duplicate, and RPS9 was used as the reference gene. In order to perform the 2^−ΔΔCt^ method, the average of the ΔCt values from Ph1 samples as a calibrator was used, to calculate the relative fold gene expression of all samples in regard to Ph1. The negative control contained everything except the cDNA. The plate was then analyzed using the Applied Biosystems™ 7500 RT-PCR system (Thermo Fisher Scientific, Waltham, MA, USA).

### 2.6. Gel Electrophoresis

The PCR products of three samples (13th, 21st, and 38th week of gestation) were stained with Sybr green solution (wells were filled with 1 µL of die and 5 µL of the PCR sample) and then placed in the wells, after which they were subjected to electrophoresis using 2% agarose gel. The electrophoresis bath had a voltage of 80 V for 50 min, while the mAs were varied depending on the current in the bath. The gel was exposed to UV light for visualization. For fragment size measurement, we used Molecular Mark (Sigma Aldrich Molecular Weight Mark 50 bp). The gel was imaged and processed using Gel Imaging Software and the Gel Doc XR + system (Bio-Rad Laboratories, Hercules, CA, USA).

## 3. Results

*DLG1* and *KIF12* expression was analyzed by microscopy on fully differentiated glomeruli (G), proximal convoluted tubules (PCTs), distal convoluted tubules (DCTs), immature glomeruli (IG), S-shaped bodies (SSBs), comma-shaped bodies (CSBs), and renal vesicles (RVs) from fetal and postnatal kidney samples. Results were expressed as percentage area and analyzed according to the five phases of kidney development as follows: Phase one (Ph1), phase two (Ph2), phase three (Ph3), phase four (Ph4), and phase five (Ph5).

### 3.1. DLG1

**Figure 1:**
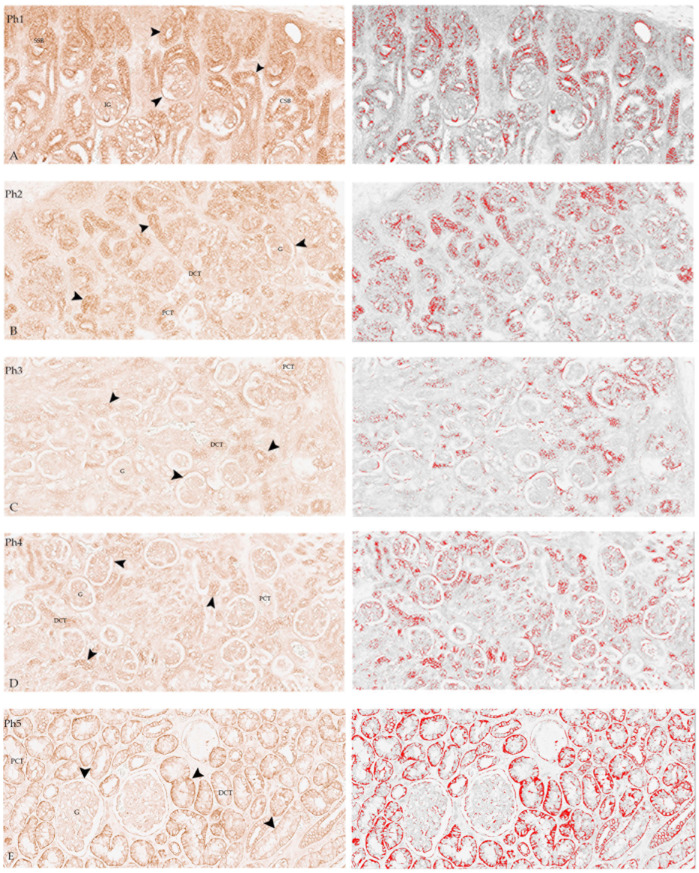
Fetal and postnatal kidneys stained with DLG1 (left), images highlighting the positive signal (red) that were used for the analysis of the area percentage of positive signal, obtained by subtraction of the median filter and thresholding (right). DLG1 (arrows) is expressed in glomeruli (G), proximal convoluted tubules (PCT), distal convoluted tubules (DCT), immature glomeruli (IG), and S-shaped bodies (SSB) in phase one (Ph1), phase two (Ph2), phase three (Ph3), phase four (Ph4), and phase five (Ph5). Ph2 to Ph4 correspond in intensity and localization of DLG1. Scale bar is 40 µm. (**A**,**B**) Individual cells in developmental structures are strongly positive; (**C**) Moderate positivity in Ph3; (**D**) Intense positive expression in G and Bowman’s capsule; (**E**) diffuse positive expression in PCTs, DCTs, and Bowman’s capsule, but weak in G (left DAB/IHC without hematoxylin counterstain, all images 20×). For negative control, see Supplementary Figure S1.

Diffuse DLG1 positive expression was found in the cell membranes of all developmental structures (Figure 1A), especially intensive in Ph1. It was found in SSBs, CSBs, and RVs, as well as in the ampullae, PCTs, DCTs, and collecting tubules. Some staining was seen in the IG, more conspicuously in Bowman’s capsule.

In Ph2 (Figure 1B), moderate membranous staining was found, mainly in the developing structures (SSBs, CSBs, and RVs), as well as PCT and DCT. The Bowman’s capsule was positive, with a lower intensity of signal in the glomeruli compared with Ph1; discrete positivity was found in the collecting ducts. 

In Ph3 (Figure 1C), a less intense signal was found than in the previous phases, though still comparable to Ph2. The staining was more intense in the nephrogenic zone, where the developmental structures (SSBs and CSBs) are located. Some staining was observed in the PCTs and DCTs and in Bowman’s capsule. A few glomeruli, predominantly immature, exhibited faint staining. 

Ph4 mostly corresponds to the second and third phase regarding the intensity and localization. Positive signals were in the PCTs, collecting tubule and the filtration membrane of the fully differentiated glomeruli and Bowman’s capsule (Figure 1D). A less intense signal was observed in the DCTs. The staining of the cells of the collecting tubules showed a more diffuse cytoplasmatic staining than in earlier phases. More intense staining was observed in the cortex than in the medulla. 

Postnatal samples showed intense staining in PCTs, DCTs, and Bowman’s capsule (Figure 1E). A signal less intense than in developing kidneys was observed in mature glomeruli.

**Figure 2:**
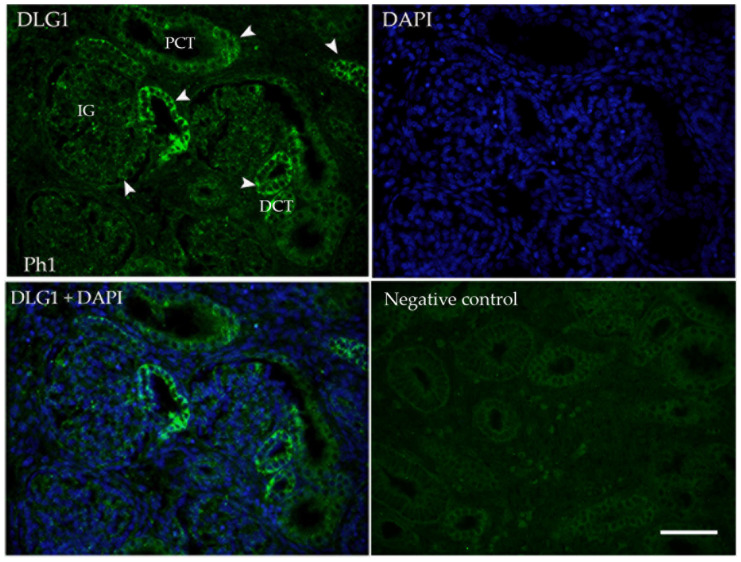
Immunofluorescence staining of human fetal kidneys (Ph1) with the DLG1 (green) and DAPI nuclear staining. Expression of DLG1 is found (arrows) in immature glomeruli (IG), proximal convoluted tubules (PCT), and distal convoluted tubules (DCT), while surrounding mesenchyme is negative. Scale bar is 50 μm.

For better subcellular localization of the positive signal, IF was performed. Signal localization and intensity corresponded to DAB/IHC, with membranous and somewhere cytoplasmatic staining through all developmental phases (Figure 2).

**Figure 3:**
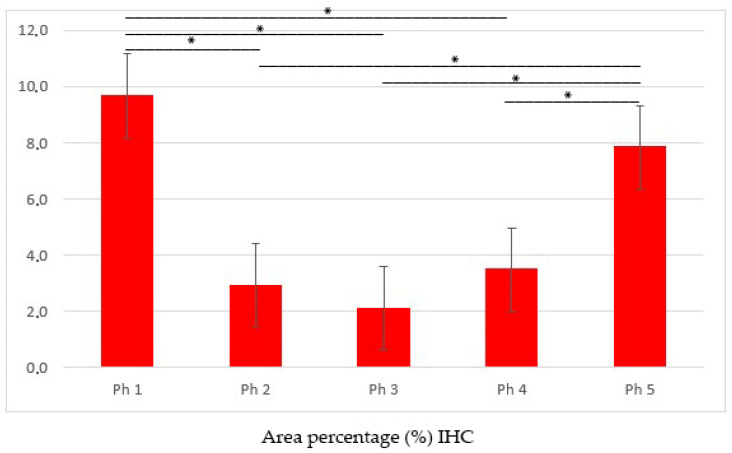
Area percentage scores of DLG1 protein in human fetal and postnatal kidneys. To quantify protein product expression, 20 non-overlapping representative visual fields per DAB-stained sample were captured. Phase one (Ph1), phase two (Ph2), phase three (Ph3), phase four prenatal (Ph4) and postnatal phase (Ph5). Data are presented as the mean ± SE (vertical line) and analyzed by the one-way ANOVA test with Tukey’s multiple comparisons test. Significant differences were indicated by * *p* < 0.0001.

In Ph1, the area percentage score was significantly higher compared to all other developmental stages (Ph2, Ph3, and Ph4) (*p* < 0.001), but corresponded to Ph5 (Figure 3). 

**Figure 4:**
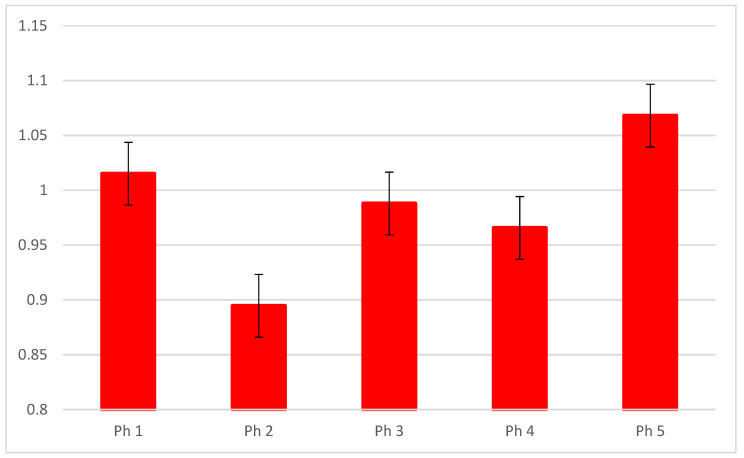
The RT-qPCR analysis of human fetal and postnatal kidneys using primers for mRNA DLG1 and their calculated 2^−∆∆Ct^.

The RT-qPCR analysis revealed the highest expression of mRNA DLG1 in Ph5 and lowest in Ph2 (Figure 4).

### 3.2. KIF12

**Figure 5:**
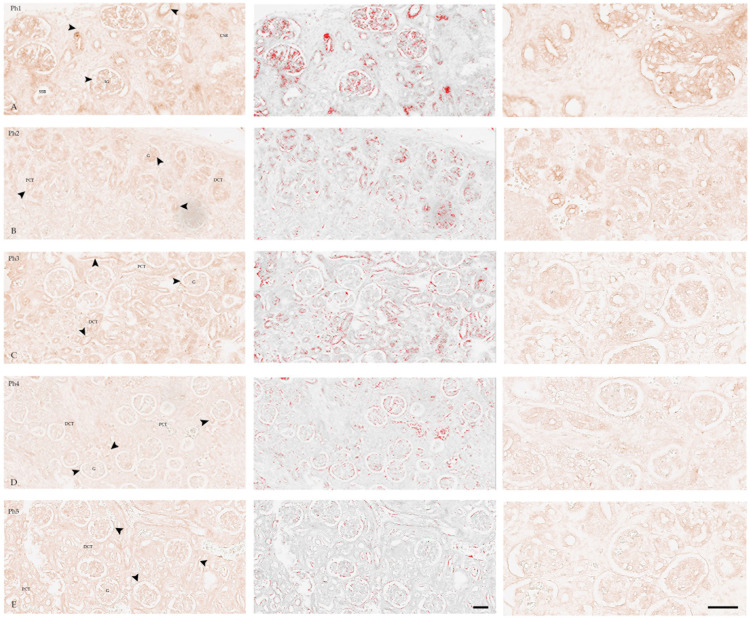
Human fetal (**A**–**D**) and postnatal (**E**) kidneys stained with KIF12 (left and right), images highlighting the positive signal (red) that were used for the analysis of the area percentage of positive signal, obtained by subtraction of the median filter and thresholding (middle). Expression of KIF12 (arrows) was seen in glomeruli (G), proximal convoluted tubules (PCTs), distal convoluted tubules (DCTs), immature glomeruli (IG), and S-shaped bodies (SSBs) in phase one (Ph1), phase two (Ph2), phase three (Ph3), phase four (Ph4), and phase five (Ph5). Developmental phases (Ph1–Ph4) mostly correspond regarding intensity and localization of KIF12. Scale bar is 40 µm. (**A**,**B**) Individual cells of IG strongly expressed KIF12; (**C**,**D**) A less intense signal can be seen in Ph3 and Ph4; (**E**) In Ph5 is seen diffuse staining of Bowman’s capsule and visceral epithelial cells, as well as luminal side of PCTs. (DAB/ICH without hemalaun counterstain, columns 1 and 2 magnification 20×, column 3 magnification 40×). For negative control, see Supplementary Figure S1.

KIF12 expression showed variable intensity in all developmental structures (Figure 5A), mostly in IG with a low intensity signal in Bowman’s capsule.

In Ph2 (Figure 5B), strong positive membranous staining was found on the luminal side of the developing structures (SSBs, CSBs, and RVs), and mild in the PCTs and DCTs, and IG including the Bowman’s capsule. 

In Ph3 (Figure 5C), a less intense, but still comparable signal was found in the G and moderate signal in the Bowman’s capsule, PCTs and DCTs. 

In Ph4, expression of KIF12 corresponded to Ph1 in intensity (Figure 5) and localization. It is found in fully differentiated G and Bowman’s capsules (Figure 5D); few PCTs and DCTs showed weak expression. 

Postnatal kidneys showed a strong positive signal in G, PCTs, DCTs, and Bowman’s capsules (Figure 5E). In tubules, the signal was located on the luminal surface. 

**Figure 6:**
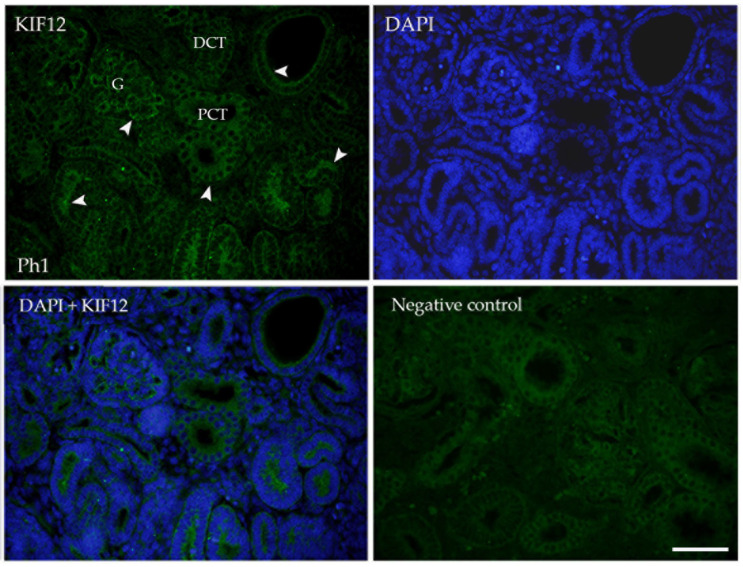
Immunofluorescence staining of human fetal kidneys (Ph1) with the KIF12 (green) and DAPI nuclear staining. Expression of KIF12 (arrows) is found in glomeruli (G), proximal convoluted tubules (PCT), and distal convoluted tubules (DCT), while the surrounding mesenchyme is negative. Scale bar is 50 μm.

The immunofluorescence signal was consistent with DAB-immunostaining in terms of intensity and localization (Figure 6).

**Figure 7:**
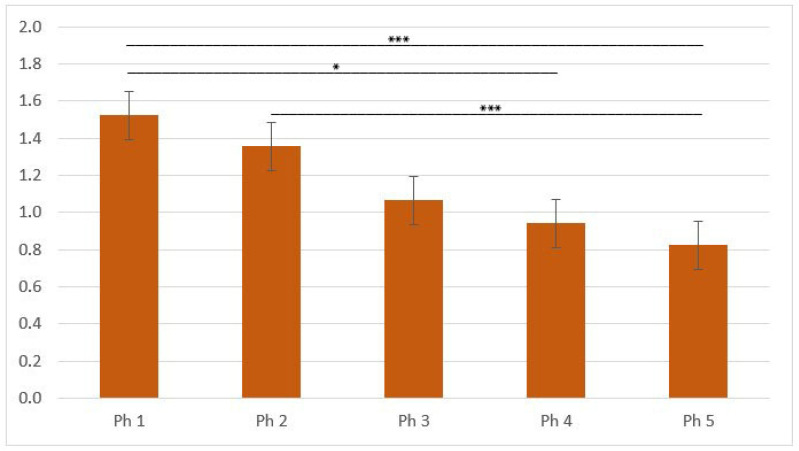
Area percentage scores of KIF12 in human fetal and postnatal kidneys. To quantify protein product expression, 20 non-overlapping representative visual fields per DAB-stained sample were captured. Phase one (Ph1), phase two (Ph2), phase three (Ph3), phase four prenatal (Ph4) and postnatal phase (Ph5). Data were presented as the mean ± SE (vertical line) and analyzed by the one-way ANOVA test with Tukey’s multiple comparisons test. Significant differences were indicated by * *p* < 0.05 and ** *p* < 0.001.

The area percentage score of the Ph1 was significantly higher than in Ph4 and Ph5 (*p* < 0.05) but corresponds to Ph2 and Ph3 in intensity of signal (Figure 7). 

**Figure 8:**
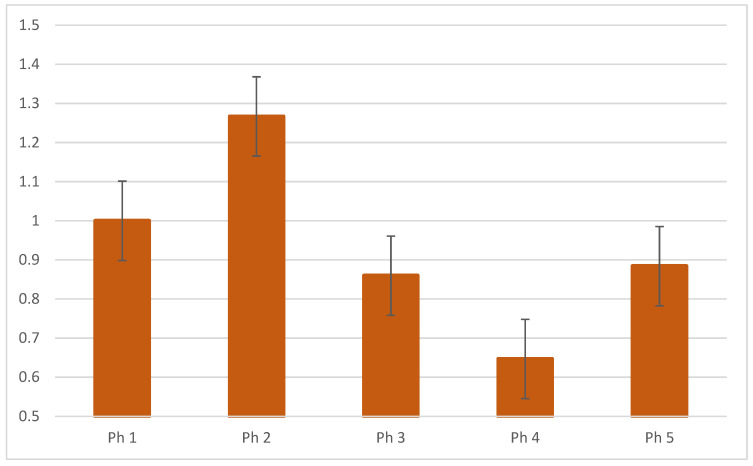
The Qrt-PCR analysis of mRNA KIF12 in human fetal and postnatal kidneys and their calculated 2^−∆∆Ct^.

The RT-qPCR analysis revealed the highest expression of mRNA KIG12 in Ph2 and lowest in Ph4 (Figure 8). 

**Figure 9:**
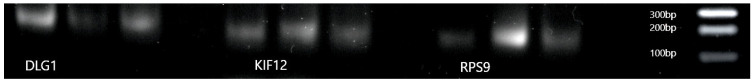
Agarose gel electrophoresis of RT-qPCR amplification products of *DLG1* (161 bp), *KIF12* (164 bp), and *RPS9* (140 bp) from 3 kidney samples.

The RT-qPCR amplification products of *DLG1*, *KIF12*, and *RPS9* were identified and visualized with gel electrophoresis in 3 samples (Figure 9).

### 3.3. Co-Localisation in Double IHC

**Figure 10:**
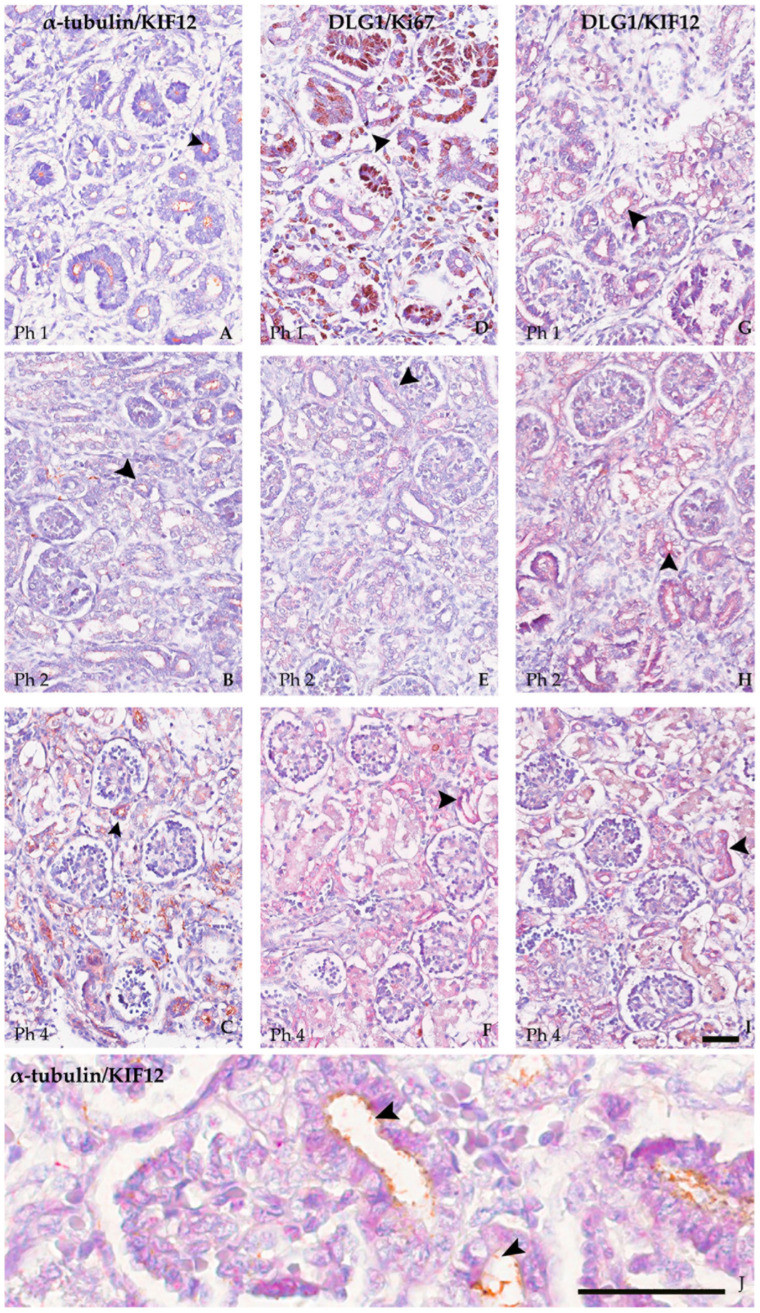
Co-localization (arrow) of KIF12 and α-tubulin, DLG1 with Ki-67 and DLG1 with KIF12 in Ph1, Ph2, and Ph4 (double immunohistochemistry, (**A**–**I**) magnification 20×, (**J**) magnification 80×, first marker red, second marker brown). Scale bar is 40 µm. For negative control, see Supplementary Figure S2.

KIF12 and α-tubulin showed strong co-localization at all developmental stages examined, predominantly on the luminal side of the tubule (Figure 10A–C). Co-localization of DLG1 and Ki-67 was also observed in the tubules, most pronounced in the Ph1, somewhat weaker in the Ph2 (Figure 10D–F). Co-localization of DLG1 and KIF12 was found occasionally, most pronounced in the tubules in Ph1 and Ph2 gestational week and weaker at Ph4, when it was detected as punctate signals in the tubules and Bowman’s capsules (Figure 10G–I).

## 4. Discussion

The aim of this study was to investigate the spatio-temporal expression of two CAKUT candidate genes, *DLG1* and *KIF12,* in rare samples of developing and postnatal human kidneys, while *α-tubulin* and *K-i67* were included because of their association with *KIF12* and *DLG1*, respectively, and due to the fact that their expression has previously been described in developing human kidneys [28,29]. Our hypothesis is that the *DLG1* and *KIF12* play a key role in kidney development, because during this period, their protein products dynamically change in time and space. The selection of the analyzed genes was based on our systematic in silico analysis to identify genetic drivers for CAKUT-associated CNVs of patients with confirmed CAKUT. In this analysis were, among others, highlighted our genes of interest as candidate genes for CAKUT [13].

Despite the great interest of other investigators in the role of *DLG1* and *KIF12* during kidney development, most previous studies have used animals or experimental in vitro models. To our knowledge, few studies have investigated the expression of *DLG1* and *KIF12* in human kidneys [13]. Our study is the first to demonstrate their expression and localization in human samples at all stages of kidney development (fetal and postnatal), as well as dynamic change in their spatio-temporal expression. Our investigation demonstrated statistically significant differences between certain developmental stages, which could help pinpoint the crucial time when *DLG1* and *KIF12* control proper renal maturation.

We confirmed *DLG1* gene expression in kidneys via quantification of relative protein and mRNA levels using DAB/IHC and IF staining, as well as RT-qPCR, respectively. We detected a strong expression of DLG1 in the developing kidney.

Previous studies in mice showed moderate expression of Dlg1 in mouse UB structures and MM. *Dlg1* knockout mice exhibited hypoplastic kidneys associated with impaired branching of the ureteric bud and decreased nephron formation [15,16,17]. The results of the aforementioned studies are in agreement with our study, which confirmed that the studied protein was present in all developmental structures arising from the UB. Furthermore, we detected protein expression in the Bowman’s capsule at all developmental stages, which is consistent with the study in a mouse model [16].

A gradual decrease in expression was observed until Ph3, when 60% of nephrons are formed [30], and then a gradual increase in Ph4 that persisted postnatally. As members of the MAGUK family, DLGs have multiple protein–protein interaction domains. Therefore, as a result, they are capable of organizing various protein complexes with a wide range of cellular functions such as regulating cell polarity in epithelia and controlling epithelial architecture by regulating mitotic spindle orientation, which is critical for cell division during tissue development [31,32,33,34,35,36]. In Ph4 and Ph5, the highest expression of DLG1 was observed in tubules. This result (with a deviation in Ph3) was confirmed by mRNA quantification. The explanation is that only interstitial growth and differentiation occurs in Ph4. During this period, the cortex increases due to the increasing length and tortuosity of the PCTs [3]. The result is a fourfold increase in glomerular filtration rate (GFR), increased tubular reabsorption of electrolytes and water, and maintenance of glomerular-tubular balance that supports the growing infant [7]. A GWAS study by Stanzick et al. that included >1.2 million individuals showed an association between a variant (rs12152266) of the *DLG1* gene and a lower estimated GFR rate. By characterizing genotype-related changes in gene expression in the human kidney, causative genes affecting renal function were identified, including *DLG1* [37]. 

The strong expression of DLG1 in Ph 4 and 5 tubules is consistent with previous studies in the developing mouse kidney, which showed expression of Dlg1 in the pretubular aggregates that later develop into renal vesicles and form proximal and distal tubules and the anlage of the loop of Henle in later differentiation steps [16].

The study by Fuja et al. showed a correlation between expression of DLG1 and Ki-67 in mammal breast carcinoma [38]. Ki-67 is a nuclear protein commonly used to detect proliferating cells and its expression is connected with cell growth [39]. In our study, DLG1 and Ki-67 were presented in the renal structures during the developmental stages. Co-localization was observed during fetal kidney development, especially in Ph1, when a more pronounced expression of Ki-67 was observed. Decrease of Ki-67 was presented during the process of development. Ki-67 expression becomes weaker, so that in the fetal kidney sample at 38 weeks of gestation, co-localization was visible only as punctate staining in the G and DCTs. This was consistent with previous studies, showing that Ki-67 expression in human nephrogenesis is highest in the early stages of metanephric glomeruli differentiation, differentiating vesicles and folding glomeruli, and decreases with glomerular maturation [29,40]. 

Given the extremely wide range of functional roles of *DLG*s in eukaryotes, our results are likely to be beneficial to future functional studies regarding this family of proteins. 

Our results confirmed *KIF12* gene expression in kidneys by several methods, including DAB/IHC, IF, and mRNA KIF12 using RT-qPCR. We detected the expression of KIF12 in tubular and glomerular cells at all developmental stages, manifested as luminal staining of tubular epithelium and membranous staining of glomerular cells. The highest expression was observed in Ph1, with a gradual decrease in the later phases and a further in the postnatal period, indicating an influence on nephrogenesis. Our previous study on human material showed strong expression of KIF12 in the UB stalk and UB-derived structures such as the epithelium of collecting ducts, whereas the surrounding mesenchyme was negative from the 6th week of development [13]. These results are consistent with our current study and confirmed that the studied proteins are present in all Ph1 developmental structures which arise from the UB. 

Recent studies detected biallelic mutations of *KIF12* in children with high GGT and cholestasis without extrahepatic abnormalities, suggesting a role in the pathogenesis of cholestatic liver disease. All *KIF12* mutated samples showed detectable protein staining in the liver samples. This suggests that nonsense mRNA decay did not occur, and a truncated protein was expressed, which may also explain the absence of symptoms in the kidney. It is believed that KIF12 modulates the expression severity of recessive homozygous biliary phenotype disease in humans [41,42]. 

The protein encoded by *Kif12* is co-localized with α-tubulin, the marker for primary cilia in large cell lines from the inner medullary collecting duct of mice [25]. Evidence suggests that primary cilia function can be impaired by dysregulation of α-tubulin, a microtubule-based organelle important for tissue homeostasis and during development, leading to cystogenesis, abnormal kidney development, and potentially chronic kidney disease [43,44,45]. Our previous study showed abnormal localization and function of primary cilia in human kidney disease associated with the development of cysts [46]. 

Additionally, our previous study analyzed the different dynamics of α-tubulin expression patterns at different stages of kidney development and showed that expression decreases when kidney tissue assumes a mature morphology, with a slight increase in expression in postnatal kidney [28]. Expression of KIF12 resembles the α-tubulin spatial-temporal expression pattern from our previous investigation [28]. In this study, we did not analyze the expression and localization of α-tubulin in all developmental stages, but found its strong association with KIF12 in several anatomical structures (SSB, CSB, PCT, DCT) and at different developmental time points.

The presence of KIF12 and co-localization with α-tubulin at all developmental stages of the human kidney, mainly on the luminal side of tubular epithelial cells, may indicate localization within primary cilia. Our results are similar to previous studies in mouse models that implicated Kif12 as a strong candidate positional gene for the major effect modifier 2 (Mpkd2) of autosomal recessive polycystic kidney disease (ARPKD) in mice. Its localization in primary cilia demonstrated that the Mpkd2 locus-associated gene encodes a primary cilia-associated protein, providing further evidence that it is a candidate gene for the Mpkd2 locus [25,47]. 

Further studies have shown that *KIF12* may be involved in the development of polycystic kidney disease. Using a functional genomics approach which involve chromatin immunoprecipitation and promoter arrays in combination with gene expression profiling, it was demonstrated that *KIF12* is the target gene of transcription factor *HNF-1β* (hepatocyte nuclear factor-1β). *HNF-1* mutations inhibited *KIF12* transcription in cultured cells and knockout mice by altering cofactor recruitment and histone modification. *KIF12* encodes a protein involved in the control of cell division and its downregulation leads to the abnormal planar cell polarity observed in cystic kidney disease [48]. 

The aforementioned studies performed in mouse models highlight the expression of *Kif12* in primary cilia, which is associated with its importance in the development of polycystic kidney disease. In our study, we detected KIF12 expression on the luminal side of tubules, co-localized with α-tubulin, a previously reported marker for primary cilia. 

Common signaling pathways have different site-and-time effects on kidney organogenesis, determining cell fate, proliferation, migration, and tissue differentiation, and overlap extensively. One of these pathways is the Sonic Hedgehog (SHH) signaling pathway controlled by a number of genes, including genes that form kidney structure and cell cycle modulators. Abnormal activation of this pathway in renal cells may represent an early event in cystogenic transformation [49,50,51]. Skalická et al. showed that SHH is the second most commonly mutated pathway in autosomal dominant PKD [52]. Several studies have shown that the KIF proteins (*KIF4*, *KIF13B*, and *KIF27*) are associated with the SHH signaling pathway which is crucial for kidney organogenesis [24,53,54]. The association of several proteins from the KIF family with the SHH signaling pathway and the detected co-localization of KIF12 with the previously reported cilia marker α-tubulin indicate a possible association of *KIF12* with the SHH signaling pathway, and thus, its possible role in regulating primary cilia formation.

In our study, the highest mRNA expression for *KIF12* was observed in the early stages of kidney development (Ph 1 and Ph2), characterized by active branching of collecting tubules, induction and progression of nephrons, which include active cell division. We believe that our results are consistent with those of previous studies that have shown that the *KIF12* gene is involved in the control of cell division [20,21,22,48].

In this study, the transcripts of *DLG1* and *KIF12* genes were quantified for the first time in the developmental stages of human kidney from week 13 to postnatal subject. The fact that *DLG1* and *KIF12* mRNA and proteins were detected at all stages of kidney development suggests their role in maintaining overall homeostasis and in the maturation of kidney structures, which continues into the postnatal period.

In mammals, *DLG1* and the kinesin family member 13B (*KIF13B*) interact directly via the guanylate kinase-like domain (GUK) of *DLG1* and an unphosphorylated MAGUK binding stalk domain of *KIF13B* with high affinity [55,56,57,58,59]. This interaction does not need the *KIF13B* to be phosphorylated and is constitutively present as long as the two proteins are in close proximity [59]. Because of this, we investigated the spatial overlap of DLG1 and KIF12 as an important paralog of KIF13B, in tissue of developing kidneys. Results revealed co-localization in the apical membrane of tubular epithelial cells. The ubiquitous expression of *KIF12* is consistent with the expression profile of *DLG1,* thus implying a functionally conserved interaction between them. Whether the possible KIF12-DLG1 complex plays a role in the regulation of cell division and/or has a hand in polarized transport of DLG1 in epithelial cells, much like the *KIF13B-DLG1* complex [55,57], is a noteworthy issue for future studies. To the best of our knowledge, this is the first report of an association between the KIF12 and DLG1 proteins. Co-localization of DLG1 and KIF12 is more pronounced at the first developmental stages, especially at the apical side of the tubular epithelial cells and in G. The expression of both proteins studied previously was highest at the first developmental stage. In the later stages, the expression of *KIF12* is extremely weak and the expression of *DLG1* predominates, resulting in weaker co-localization visible as a punctate staining within G and tubules. Characterization of the supposed *KIF12-DLG1* complex might be necessary for describing the physiological basis of *DLG1* in motile functions and cellular transport during kidney development.

The spatial and temporal events leading to CAKUT are critical to the resulting phenotypes. A problem that occurs early in nephrogenesis causes a severe defect, whereas defects that occur later are generally less severe. Since our study showed that the mRNA for *DLG1* and *KIF12* is expressed at the earliest stages, we consider this as further evidence that both genes are strong CAKUT candidate genes.

The limitation of our study was the small number of samples, which were difficult to obtain because fetal human samples are rare. We also stained 3 representative samples from three different developmental stages for co-localization studies that did not cover all developmental stages analyzed in IHC single staining. In addition, the specimens were not fresh but were fixed in formalin and paraffin embedded.

In conclusion, kidney development is a dynamic process influenced by many factors. If the conditions are not met, suboptimal development in turn lead to the diseases, either intrauterine or in the postnatal period. Signaling pathways and consequently, the expression of cellular proteins are in dynamic and flexible changes. The input signals depend on extracellular stimuli, the cell proliferation and differentiation, spatial-temporal aspects, and metabolic and physiological state of the cells. Our study showed significant differences in the spatiotemporal expression of *DLG1* i *KIF12* in prenatal and postnatal development. Renal expression patterns of their proteins could be associated with impaired renal function, leading to congenital diseases and chronic renal failure.

## Figures and Tables

**Table 1:** Primary antibodies used for Immunohistochemistry.

Gene	Primary Antibody
*DLG1*	Mouse anti-DLG1 sc-9961, 1:50; Santa Cruz, CA, USA
*KIF12*	Mouse anti-KIF12 sc-376766, 1:50; Santa Cruz, CA, USA
*α-tubulin*	Rabbit anti-α-tubulin ab179484, 1:50, Abcam, Cambridge, UK
*Ki-67*	Mouse anti-Ki-67 M7240, 1:100, Agilent Technologies, CA, USA

**Table 2:** Primers used in RT-qPCR.

Gene	Forward Primer	Reverse Primer
*DLG1*	5′-TCC ACC CAG GCA AAT CCT CC-3′	5′-GGG TTG TCC GTA CCT CCT GC-3′
*KIF12*	5′-CCG GAC TCT GCA GTT TCT CCT G-3′	5′-GGC GAA GGT CCT CTG CAT GAT-3′
*RPS9*	5′-GGA TTT CTT AGA GAG ACG CCT G-3′	5′-GGA CAA TGA AGG ACG GGA TG-3′

## Data Availability

Data are available per request.

## Supplementary Materials

The following supporting information can be downloaded at: https://www.mdpi.com/article/10.3390/biom13020340/s1, Figure S1: Negative control for IHC staining, human fetal (Ph1-4) and postnatal (Ph5) kidneys. Magnification 20×. Scale bar is 40 µm.; Figure S2: Negative control for co-localization double immunohistochemistry staining, human fetal (Ph1-3) kidneys. Magnification 20×. Scale bar is 40 µm.
