# Supplementary material for: Spatio-Temporal Expression Pattern of CAKUT Candidate Genes DLG1 and KIF12 during Human Kidney Development
Source: Biomolecules. 2023 Feb 9;13(2):340. doi: 10.3390/biom13020340 (PMC9953652; doi:10.3390/biom13020340)
Supplement: Supplementary file 1 [file biomolecules-13-00340-s001.zip › biomolecules-2149424-supplementary.pdf]

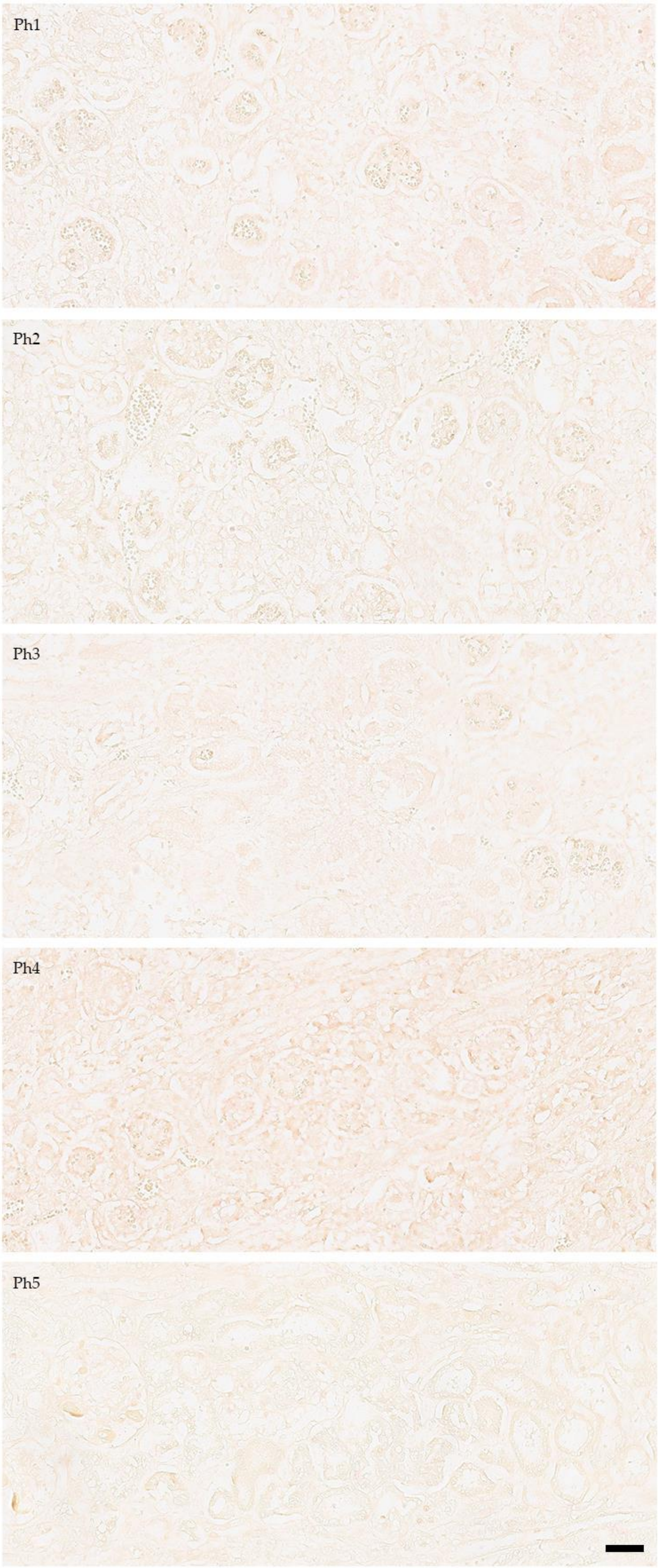

Supplementary Figure S1. Negative control for IHC staining, human fetal (Ph1-4) and postnatal (Ph5) kidneys (for Figures 1 and 5). Magnification 20x. Scale bar is 40 μm.

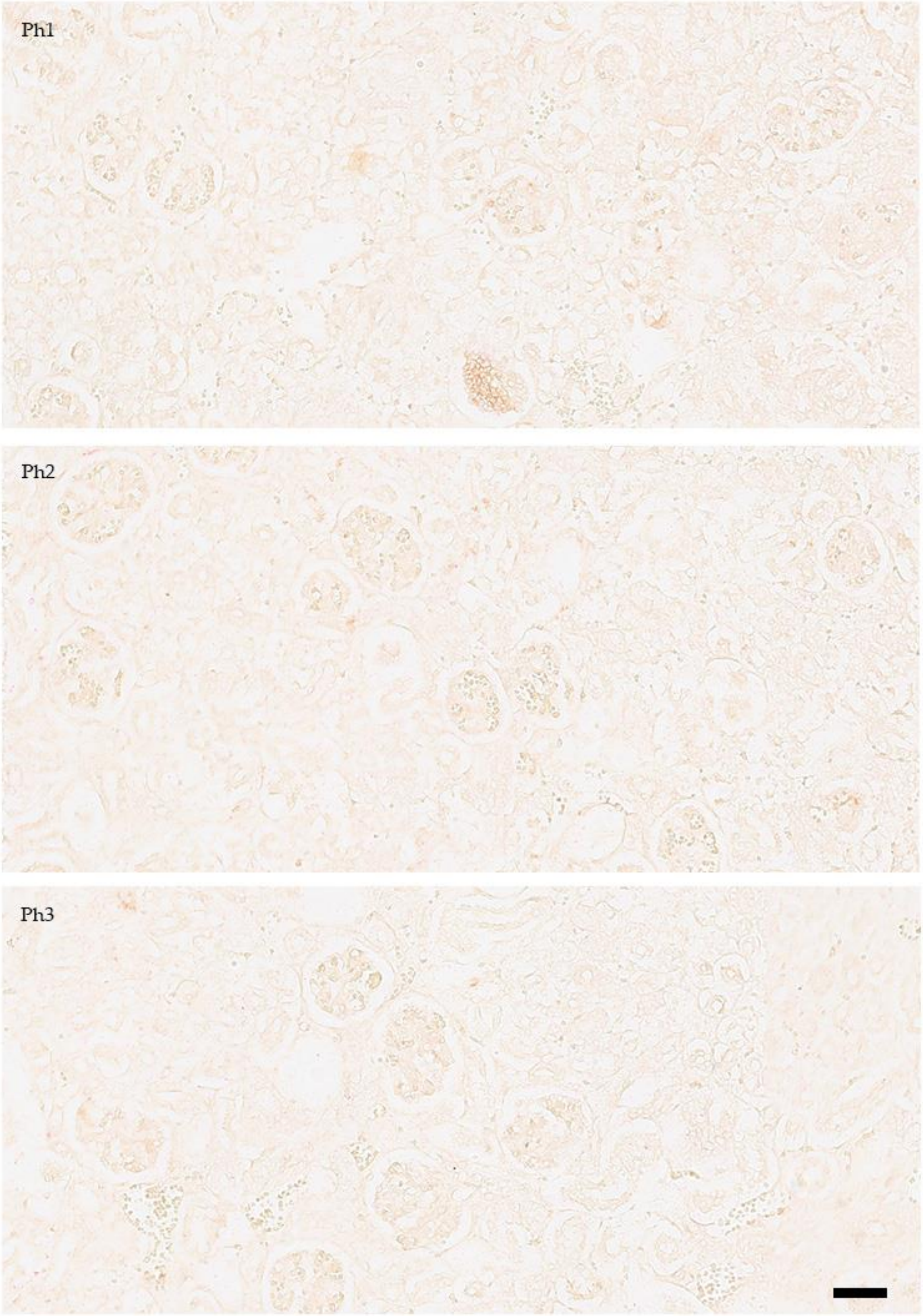

Supplementary Figure S2. Negative control for co-localization double immunohistochemistry staining, human fetal (Ph1-3) kidneys (for Figure 10). Magnification 20x. Scale bar is 40 μm.
